# Integrative Bioinformatics and Machine Learning Identify Novel Diagnostic Biomarkers and Molecular Mechanisms in Sjögren’s Syndrome

**DOI:** 10.1155/ijog/5044551

**Published:** 2026-01-16

**Authors:** Hua Xu, Yong Liu, Yuyin Song, Yifan Zheng, Haifeng Jing, Yanfei Gao, Depeng Zhou, Xiang Chi, Jia Chen

**Affiliations:** ^1^ Department of Laboratory Medicine, Panjin Liaoyou Gem Flower Hospital, Panjin, Liaoning, China; ^2^ Department of General Practice, Panjin Liaoyou Gem Flower Hospital, Panjin, Liaoning, China

**Keywords:** CIBERSORT, diagnostic biomarkers, immune microenvironment, machine learning, Sjögren’s syndrome

## Abstract

**Background:**

Sjögren’s syndrome (SS) is a chronic autoimmune disorder characterized by significant diagnostic challenges due to nonspecific symptoms and a lack of reliable biomarkers, often resulting in delayed diagnosis and suboptimal patient management.

**Objective:**

This study is aimed at identifying novel diagnostic biomarkers and elucidating the molecular mechanisms underlying SS pathogenesis through integrative bioinformatics and machine learning approaches.

**Methods:**

We analyzed three peripheral blood transcriptomic datasets (GSE51092, GSE66795, and GSE84844) comprising a total of 351 SS patients and 91 healthy controls. Differential expression analysis, weighted gene coexpression network analysis (WGCNA), and 12 machine learning algorithms were employed to identify robust diagnostic biomarkers. Immune cell infiltration was assessed using CIBERSORT, and single‐cell RNA sequencing data (GSE157278) were analyzed to validate cell‐type‐specific expression patterns. Drug repurposing analysis was conducted using the L1000FWD platform.

**Results:**

We identified 12 hub genes (EPSTI1, IFIH1, CXCL10, TNFSF10, GBP5, PARP9, IFI44, LAP3, IFIT2, IFI44L, PARP12, and OAS1) with exceptional diagnostic performance (AUC = 0.994 in training, 0.838 in internal validation, and 0.825 in external validation). These biomarkers showed significant correlations with clinical indicators including ANA, Ro/SSA, and La/SSB (*p* < 0.05). Immune‐infiltration analysis revealed pronounced immune dysregulation in SS patients, characterized by an imbalance between naive and memory B cells and reduced CD8^+^ T cells and regulatory T cells (Tregs). Single‐cell transcriptomics confirmed predominant expression in monocytes and dendritic cells, with additional significant expression in B cells and CD4^+^ T cells. Virtual knockdown analysis implicated these genes in antigen presentation, interferon signaling, and leukocyte trafficking. Drug repurposing identified FDA‐approved candidates such as nisoldipine and exemestane as potential therapeutics.

**Conclusion:**

Our integrative approach identifies 12 robust diagnostic biomarkers for SS, offering new insights into disease mechanisms and highlighting potential therapeutic targets for this challenging autoimmune disorder.

## 1. Introduction

Sjögren’s syndrome (SS) is a systemic autoimmune disease characterized by chronic exocrine gland dysfunction, primarily affecting lacrimal and salivary glands, with typical clinical manifestations including dry mouth and dry eyes [1–3]. However, SS demonstrates significant heterogeneity, often presenting with systemic symptoms such as fatigue and musculoskeletal pain beyond localized glandular involvement, and may involve multiple organ systems including the liver, lungs, kidneys, and nervous system. A subset of patients may progress to B cell lymphoma [1–3]. Epidemiological surveys reveal geographical and ethnic variations in global SS prevalence (0.03%–5%), showing an upward trend with significantly higher incidence in females compared to males [4–7]. Insufficient public awareness of SS and its nonspecific symptoms contribute to persistently high misdiagnosis rates [4, 5].

Regarding pathogenesis, current evidence suggests that SS arises from aberrant immune system activation in genetically susceptible individuals following environmental triggers, wherein B cells orchestrate multifaceted immunopathological mechanisms through costimulatory molecules (such as CD226), participating in processes including autoantibody production, antigen presentation, and cytokine secretion. However, the precise molecular mechanisms remain incompletely elucidated [8, 9]. In clinical practice, the insidious onset of early symptoms and inadequate sensitivity of existing biomarkers often lead to delayed diagnosis until extraglandular organ involvement occurs, highlighting the urgent need for novel diagnostic tools [10]. Current SS diagnosis relies on comprehensive evaluation of clinical symptoms, physical signs, histopathology, and serological testing. While traditional serological markers including anti‐SSA/Ro, anti‐SSB/La antibodies, antinuclear antibodies (ANAs), and rheumatoid factor (RF) play crucial roles in diagnosis and differential diagnosis, their high false‐negative rates in anti‐SSA/SSB‐negative patients significantly complicate clinical diagnosis [11–15]. This diagnostic dilemma has driven researchers to actively employ high‐throughput omics technologies in the continuous exploration of novel molecular biomarkers.

Recent advances in machine learning (ML)–based multiomics integration analysis have provided new perspectives for SS precision diagnosis. However, substantial heterogeneity in study cohort selection, analytical methodologies, and validation approaches among existing studies has limited the generalizability of research findings and hindered clinical translation [16–19]. To address these challenges, we constructed a computational framework integrating 113 ML model configurations. Leveraging three independent peripheral blood transcriptomic cohorts from the GEO database, we systematically screened for diagnostically valuable molecular markers, characterized the SS immune microenvironment with CIBERSORT, and introduced single‐cell RNA‐seq data to validate the expression and regulatory networks of core genes at the cellular level. The goal was to establish a highly sensitive and specific diagnostic model while uncovering novel molecular targets that illuminate SS pathogenesis.

## 2. Methods

### 2.1. Data Acquisition and Preprocessing

In this study, we obtained three peripheral blood transcriptomic datasets from the Gene Expression Omnibus (GEO) database: GSE51092 (Nss = 190, Nnormal = 32) [20], GSE66795 (Nss = 131, Nnormal = 29) [21], and GSE84844 (Nss = 30, Nnormal = 30) [22]. To corroborate findings at single‐cell resolution, we also included the publicly available scRNA‐seq dataset GSE157278, which profiled peripheral blood mononuclear cells (PBMCs) from five pSS patients and five healthy controls [23].

After integrating GSE66795 and GSE84844 (Nss = 161, Nnormal = 59), batch effects were removed with the ComBat algorithm (sva package) [24], and correction quality was verified by principal component analysis (PCA). The merged data were then randomly split 7:3 into a discovery cohort (Nss = 112, Nnormal = 41) and an internal validation cohort (Nss = 49, Nnormal = 18), whereas GSE51092 served as an independent external validation set (Table 1).

**Table 1:** Basic information of GEO datasets used in the study.

**ID**	**GSE series**	**Disease**	**Samples**	**Source types**	**Platform**	**Country**
1	GSE66795	SS	131 SS patients and 29 normal controls	Whole blood	GPL10558	United Kingdom
2	GSE84844	SS	30 SS patients and 30 normal controls	Whole blood	GPL570	Japan
3	GSE51092	SS	190 SS patients and 32 normal controls	Whole blood	GPL6884	USA
4	GSE157278	PSS	5 pSS patients and 5 normal controls	Peripheral blood mononuclear cells	GPL24676	China

### 2.2. Screening of Differentially Expressed Genes (DEGs)

DEGs across datasets were analyzed using the robust rank aggregation (RRA) algorithm combined with the limma package [25]. The screening criteria were set as an adjusted *p* value < 0.05 and |log2(fold change)| > 0.585, ultimately identifying DEGs associated with SS.

### 2.3. Weighted Gene Coexpression Network Analysis (WGCNA)

WGCNA [26] was performed on the discovery cohort to construct a gene coexpression network. A scale‐free topology criterion determined the soft threshold power (*β* = 10). The adjacency matrix was converted into a topological overlap matrix (TOM), and gene modules were identified using dynamic tree cutting. Module–trait relationships were calculated to identify SS‐associated modules.

### 2.4. Identification of Key Genes and Functional Enrichment

Key SS‐related genes were selected by intersecting WGCNA module genes with DEGs using Venn diagrams. Functional enrichment analysis (Gene Ontology [GO] and KEGG pathways) was conducted via the clusterProfiler package, with significance thresholds set at FDR < 0.05 and *p* < 0.05.

### 2.5. Diagnostic Model Construction and Validation

To ensure robust and reproducible selection of diagnostic biomarkers, we developed a comprehensive ML framework that integrates multiple feature selection and classification algorithms. Twelve distinct algorithms were employed: Lasso regression, ridge regression, elastic net (Enet), stepwise generalized linear model (Stepglm), support vector machine (SVM), random forest (RF), extreme gradient boosting (XGBoost), generalized linear model boosting (glmBoost), partial least squares generalized linear model (plsRglm), gradient boosting machine (GBM), naïve Bayes, and linear discriminant analysis (LDA). To maximize feature selection robustness, Stepglm was applied in three directions—forward, backward, and bidirectional—and all algorithmic variants were systematically paired, yielding 113 unique model architectures for comprehensive evaluation.

Hyperparameter optimization was performed independently for each algorithm through grid search coupled with 10‐fold cross‐validation to minimize overfitting and enhance generalizability. The regularization parameter *λ* for Lasso and Ridge regression was selected according to minimum cross‐validated deviance. For Enet, the mixing parameter *α* was tuned from 0.1 to 0.9 in increments of 0.1. RF was configured with 1000 trees and a terminal node size of 5; variable importance was estimated via permutation. XGBoost parameters—maximum depth (2), learning rate (*η* = 1), and number of boosting rounds—were optimized by internal fivefold cross‐validation. GBM was trained with an initial 10,000 trees, interaction depth of 3, and shrinkage of 0.001; the optimal number of iterations was determined by minimizing cross‐validated error. Stepwise feature selection was guided by the Akaike Information Criterion (AIC).

All models were trained on normalized transcriptomic data from the discovery cohort, with consistent centering and scaling applied to both training and validation sets. Model performance was evaluated primarily using the area under the receiver operating characteristic curve (AUC); classification accuracy served as a secondary metric.

Protein–protein interaction networks were constructed using GeneMANIA [27] (https://genemania.org) to explore functional relationships among selected biomarkers.

### 2.6. Correlation With Clinical Indicators

Spearman’s rank correlation analysis assessed associations between biomarkers and clinical indicators (ANA, anti‐Ro/SSA, and anti‐La/SSB) in the GSE84844 dataset. Significance was defined as *p* < 0.05.

### 2.7. Immune Cell Infiltration Analysis

CIBERSORT [28] quantified 22 immune cell subtypes in SS samples. Violin plots visualized differential immune cell proportions, and Spearman correlations linked biomarkers to immune cell infiltration levels (*p* < 0.05).

### 2.8. Single‐Cell RNA Sequencing Data Processing and Annotation

To resolve cell‐type‐specific expression of the 12 hub genes, we analyzed publicly available scRNA‐seq data of PBMCs from SS patients and healthy controls (GSE157278). After quality control (nFeature_RNA > 200, percent.mt < 10*%*, percent.hb < 3*%*, and percent.platelet < 1*%*), we normalized the data with SCTransform, performed batch correction using Harmony, and carried out unsupervised clustering. Cell identities were annotated using SingleR with multiple immune reference datasets (Human Primary Cell Atlas, Monaco Immune Data, Blueprint/ENCODE blood atlas, DICE database, and a comprehensive human hematopoietic compendium). Hub gene expression across cell types and disease states was compared using the Wilcoxon rank‐sum test (SS vs. HC, |log2FC| > 0.5, FDR < 0.05, Benjamini–Hochberg correction).

### 2.9. Virtual Knockdown and Gene Regulatory Network Analysis

To infer functional impact of hub genes, we performed virtual knockdown with scTenifoldKnk on a curated gene set (union of highly variable genes +12 hub genes). For each target gene, we removed it from the network, recomputed edge weights, and ranked downstream perturbations by composite score (|*Z* score| × network distance). The Top 20 perturbed genes were subjected to GO biological process (BP) enrichment with clusterProfiler (*p* < 0.05).

### 2.10. Drug Repurposing Screening

The L1000FWD platform [29] (https://maayanlab.cloud/l1000fwd) was used for reverse drug screening. Candidate compounds with inverse correlations to the 12‐core‐gene expression profile were prioritized.

## 3. Results

### 3.1. Identification of DEGs

We performed differential expression analysis on three independent microarray datasets (GSE51092, GSE66795, and GSE84844) using the limma package. Applying thresholds of adjusted *p* < 0.05 and |log2FC| > 0.585, DEGs were identified in each dataset, with their distributions were visualized via volcano plots (Figures 1a, 1b, and 1c). Subsequent cross‐cohort consistency analysis using the RRA algorithm identified 50 high‐confidence DEGs, including 48 upregulated and 2 downregulated genes. The expression patterns of these DEGs were illustrated in a heat map (Figure 1d), establishing a foundation for downstream analyses.

Figure 1Screening of key DEGs between SS patients and controlgroups. (a) Volcano plot showing DEGs obtained in the GSE51092 dataset, with red and green colors indicating up‐ and downregulated genes, respectively. (b)Volcano plot showing DEGs obtained in the GSE66795 dataset, with red and green colors indicating up‐ and downregulated genes, respectively. (c) Volcano plot showing DEGs obtained in the GSE84844 dataset, with red and green colors indicating up‐ and downregulated genes, respectively. (d) RRA integration analysis of DEGs with up‐ (red) and downregulated (green) genes obtained from GSE51092, GSE66795, and GSE84844 datasets.

**Figure:**
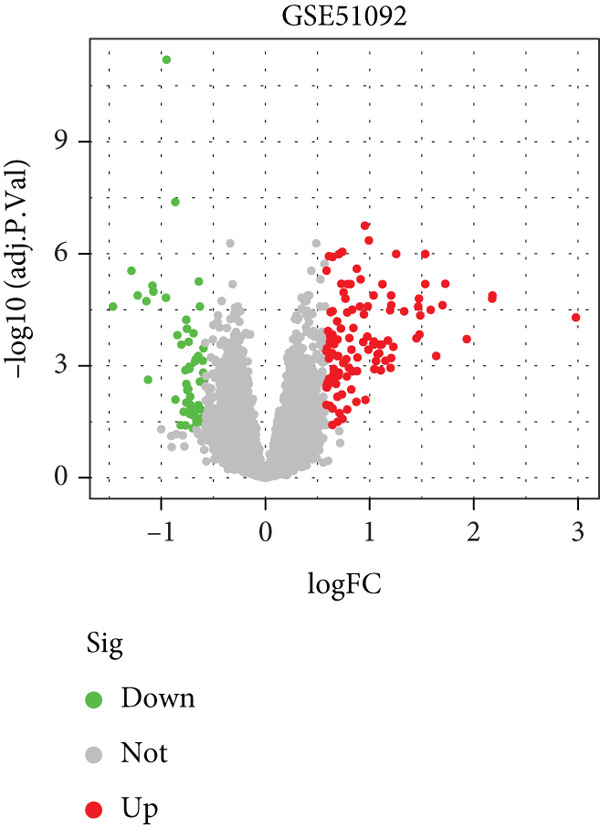
(a)

**Figure:**
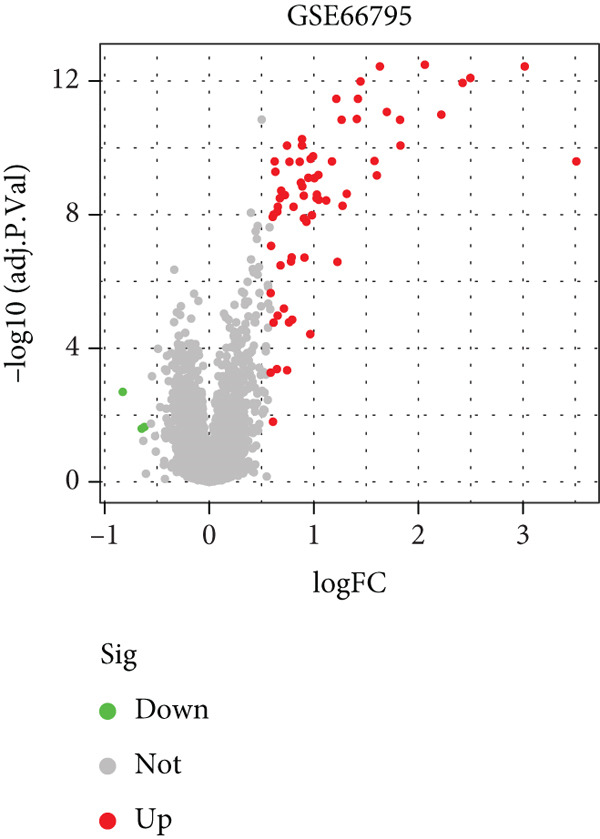
(b)

**Figure:**
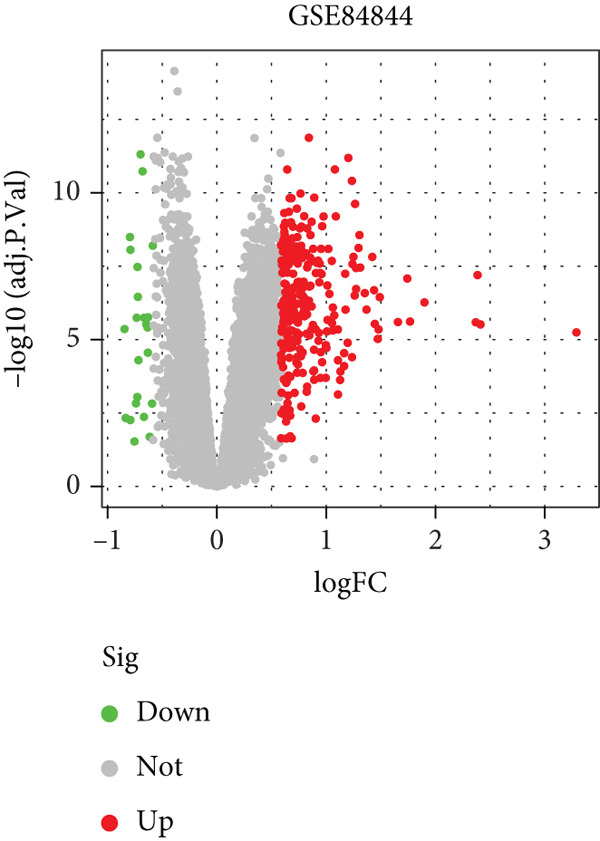
(c)

**Figure:**
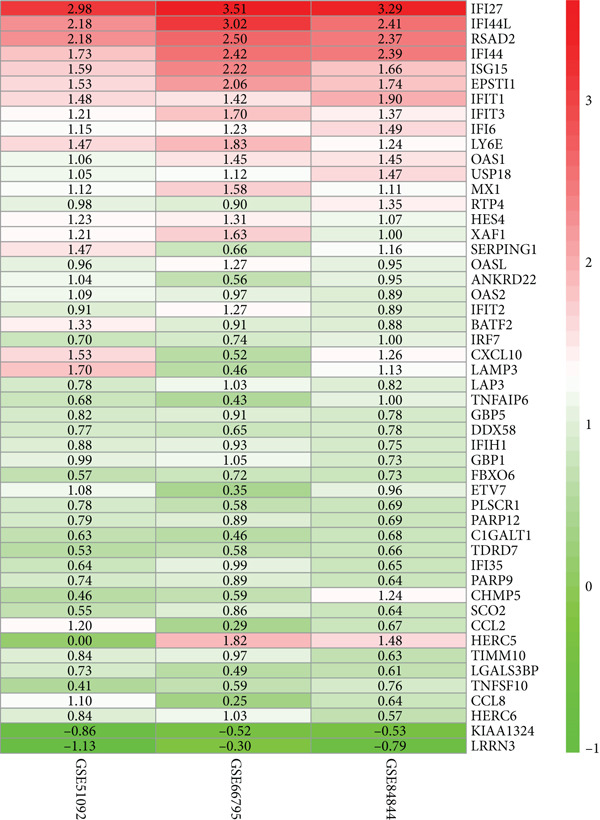
(d)

### 3.2. Dataset Integration and Partition

Two SS‐related gene expression datasets (GSE66795 and GSE84844) were integrated after batch effect correction via the ComBat algorithm, forming a standardized cohort (Nss = 161, Nnormal = 59). PCA demonstrated that batch effects were effectively removed, as sample clustering was no longer influenced by dataset origin (Figure 2). The corrected data were randomly divided into a discovery cohort (Nss = 112, Nnormal = 41) and an internal validation cohort (Nss = 49, Nnormal = 18) at a 7:3 ratio for model development and validation.

Figure 2Integration of SS datasets. (a) PCA of two original SS datasets prior to batch effect correction. (b) PCA of integrated SS dataset after batch effect correction.

**Figure:**
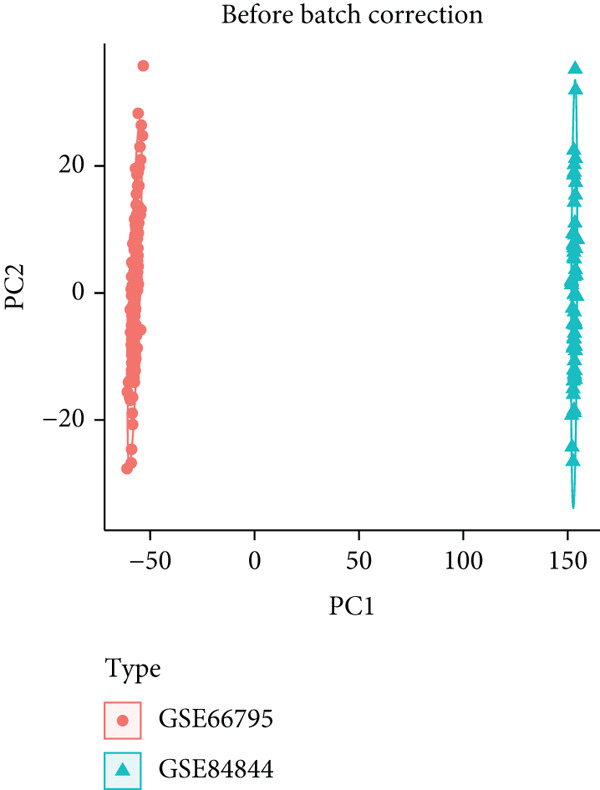
(a)

**Figure:**
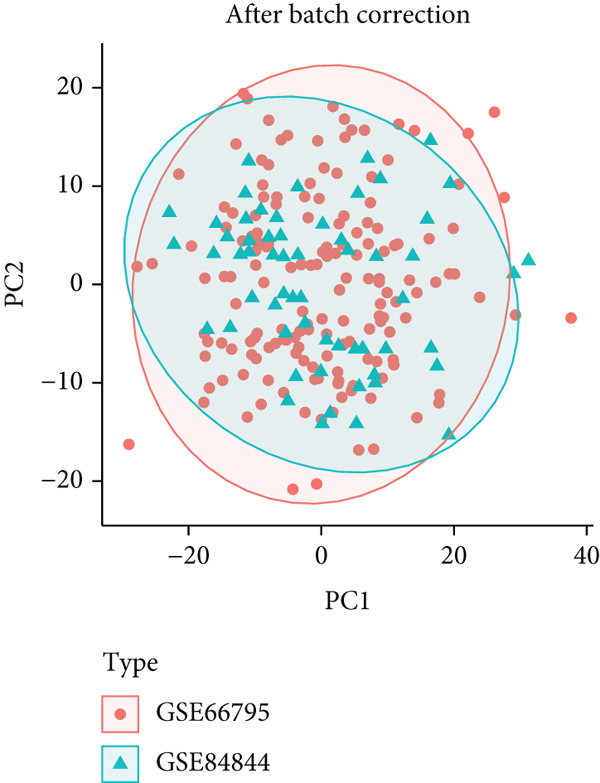
(b)

### 3.3. Weighted Gene Coexpression Network Construction and Key Module Identification

A weighted gene coexpression network was constructed using transcriptomic data from the discovery cohort to elucidate systemic biological features of gene expression regulation. The optimal soft threshold (*β* = 10) was determined by scale‐free topology fit analysis (*R* ^2^ = 0.863) (Figure 3a). Dynamic tree‐cutting with a merge threshold of 0.25 yielded four distinct modules: blue (*n* = 85), brown (*n* = 68), gray (*n* = 124), and turquoise (*n* = 85). Notably, the brown module exhibited the strongest correlation with SS (*r* = 0.58, *p* = 2e − 15, Figure 3b). Moreover, the scatter plot of gene significance versus module membership for the brown module displayed a significant correlation (cor = 0.83, *p* = 2.2e − 18, Figure 3c), suggesting its pivotal role in SS pathogenesis and highlighting it as a key target for functional studies.

Figure 3WGCNA identification of gene modules associated with SS. (a) Choosing the best soft‐threshold power. (b) The heat map of module trait relationships shows associations of various gene modules with clinical status. Red and blue cubes indicate positive and negative correlations, respectively. The consensus correlation between modules and phenotypes was presented as a number in every cube, with *p* values in parentheses. (c) Scatter plot showing the relationship between gene significance and module membership in the brown module.

**Figure:**
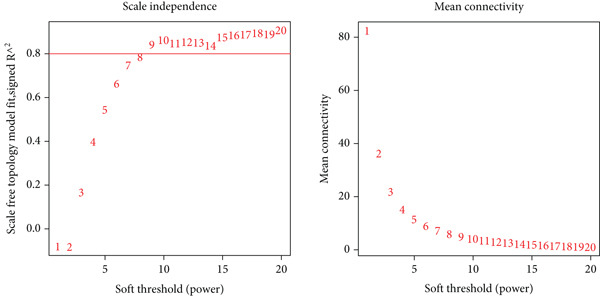
(a)

**Figure:**
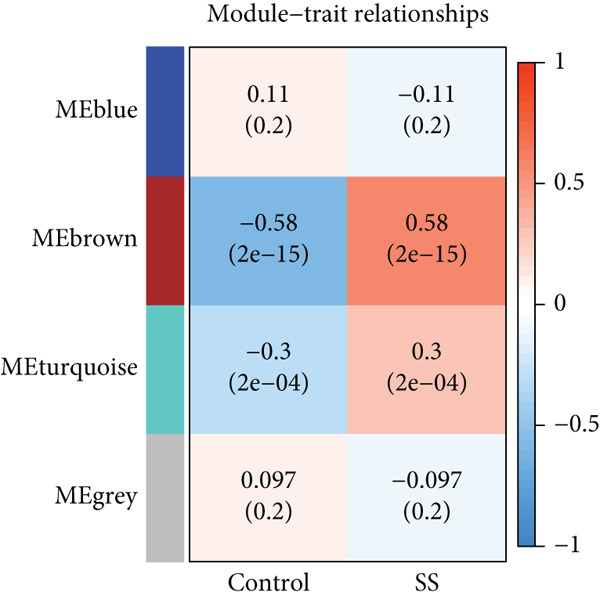
(b)

**Figure:**
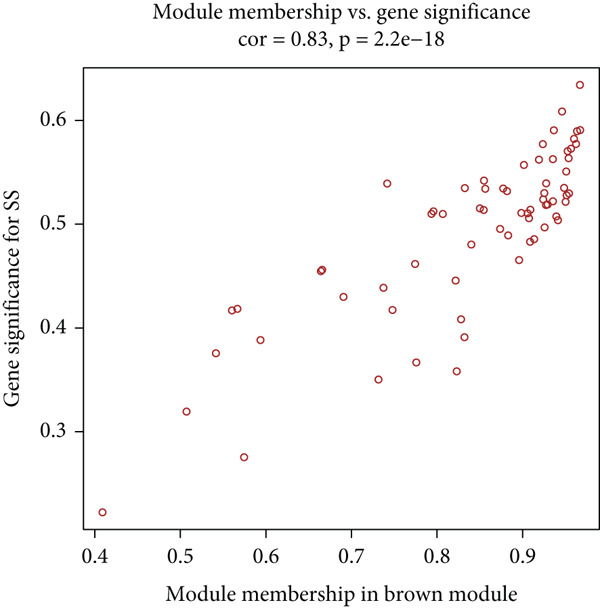
(c)

### 3.4. Screening and Enrichment Analysis of SS‐Associated Genes

Intersection analysis between the brown module genes and DEGs identified 43 candidate SS‐associated genes (Figure 4a). GO enrichment revealed significant associations with BPs such as response to virus, defense response to symbiont, and regulation of viral life cycle. Cellular component (CC) terms included mitochondrial inner membrane, collagen‐containing extracellular matrix, and organelle outer membrane. Molecular function (MF) terms were enriched in double‐stranded RNA binding, GTP binding, and nucleotidyltransferase activity (Figure 4b). KEGG pathway analysis further highlighted enrichment in Influenza A, Hepatitis C, COVID‐19, and RIG‐I‐like receptor signaling pathway (Figure 4c), implicating virus‐induced dysregulation of innate immunity in SS pathogenesis.

Figure 4Screening and enrichment analysis of SS‐associated genes. (a) Venn plot showing the intersecting genes between MEbrown module and DEGs associated with SS. (b) The Top 10 items of the GO enrichment analyses are illustrated as a bubble plot. BP, biological process; CC, cellular component; MF, molecular function. (c) Circos plot of the KEGG pathway enrichment results. The inner red circle represents the *z*‐score values, and the outer circle represents the number of genes enriched in the pathway.

**Figure:**
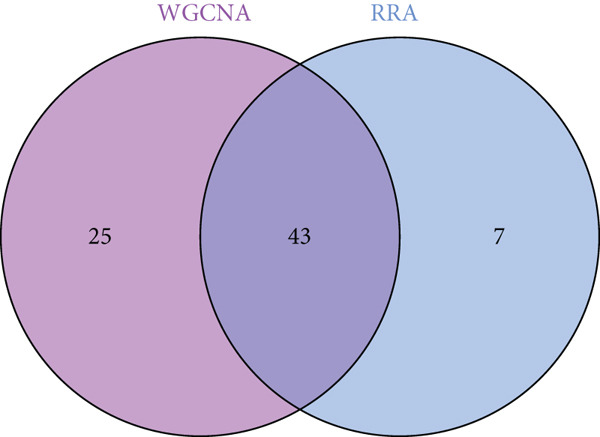
(a)

**Figure:**
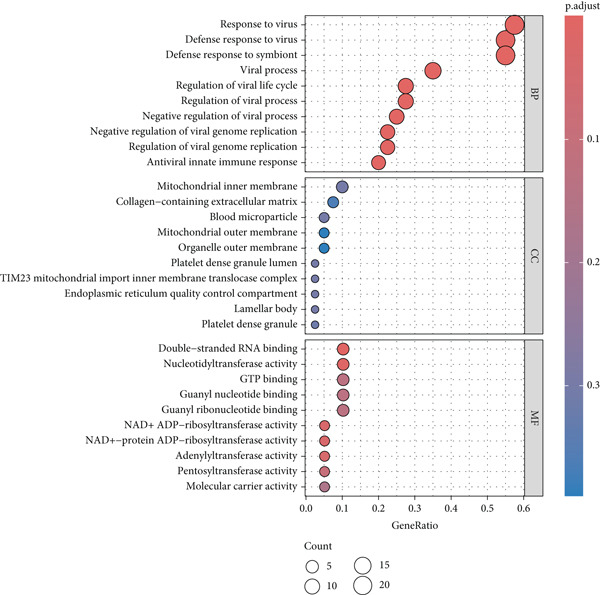
(b)

**Figure:**
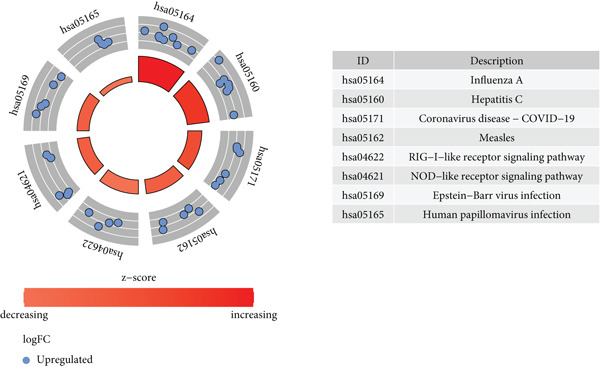
(c)

### 3.5. Identification of Diagnostic Hub Genes via ML

To screen for precise diagnostic biomarkers for SS, we applied an integrative ML strategy to the 43 candidate genes. After systematically evaluating 12 algorithms with 10‐fold cross‐validation, a composite model combining Stepglm (both) and RF demonstrated optimal diagnostic performance. This model identified 12 core genes (EPSTI1, IFIH1, CXCL10, TNFSF10, GBP5, PARP9, IFI44, LAP3, IFIT2, IFI44L, PARP12, and OAS1), which exhibited exceptional discriminatory power in the training cohort (AUC = 0.994). Robust performance was maintained in the validation cohorts, with an AUC of 0.838 in the internal validation set and 0.825 in the external validation set (Figures 5a, 5b, 5c, and 5d). We further determined a unified classification threshold of 0.6776 by maximizing the Youden index on the training set. At this threshold, the model achieved a sensitivity of 92.0%, a specificity of 100.0%, and a balanced accuracy of 96.0% in the training set. It also maintained robust performance in the overall validation set (*n* = 289), yielding a sensitivity of 70.7%, a specificity of 84.0%, and balanced accuracy of 77.4% (Supporting Information 1: Table S1). All 12 biomarkers were significantly upregulated in SS patients compared to healthy controls (Wilcoxon rank‐sum test, *p* < 0.05; Figure 5g). Notably, each gene also demonstrated considerable standalone diagnostic potential, with individual AUCs exceeding 0.8 (Figure 5e).

Figure 5Selection of hub genes associated with SS through machine learning and construction of a diagnostic model. (a) 113 machine learning algorithm combinations evaluated via 10‐fold cross‐validation. (b) ROC curve of the model in the training cohort. (c) ROC curve of the model in the internal validation cohort. (d) ROC curve of the model in the external validation cohort. (e) ROC curve for the 12 hub genes in the training cohort. (f) GeneMANIA network analysis reveals functional interactions among the core genes. (g) Expression of the core genes in the SS and control groups.

**Figure:**
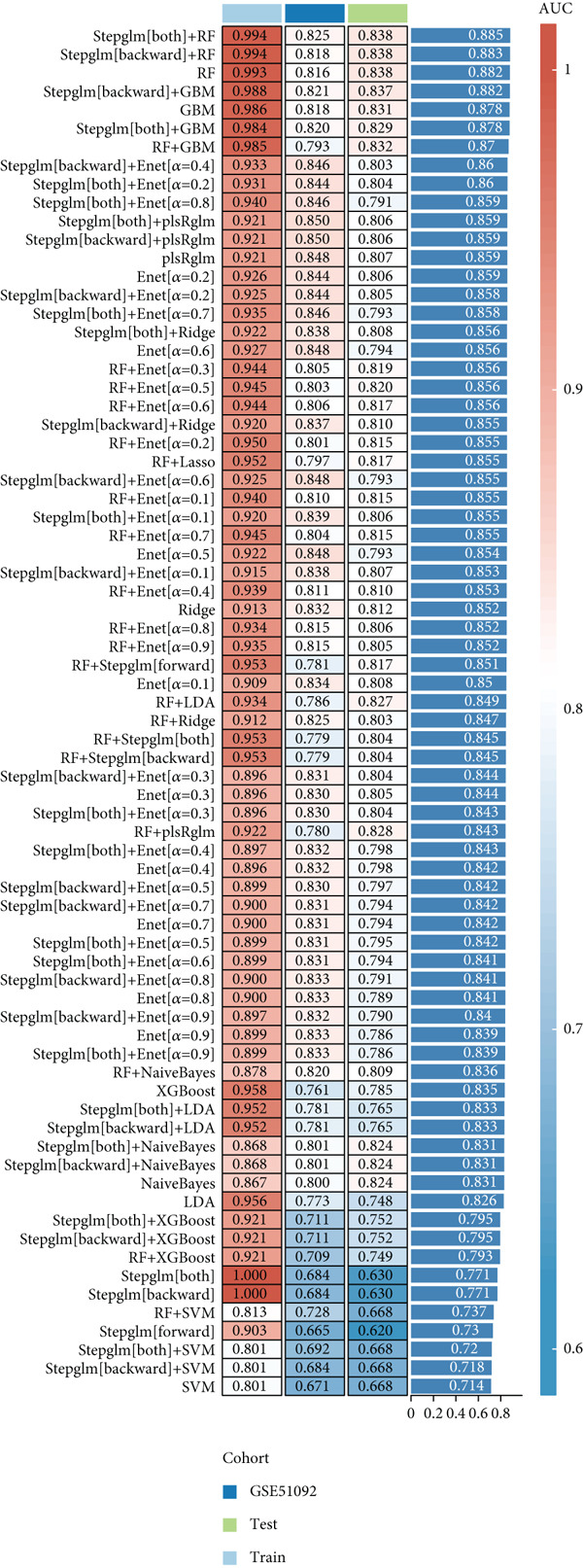
(a)

**Figure:**
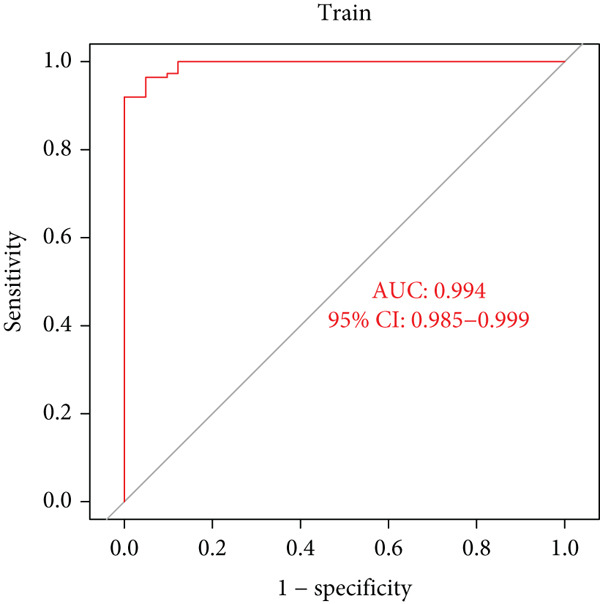
(b)

**Figure:**
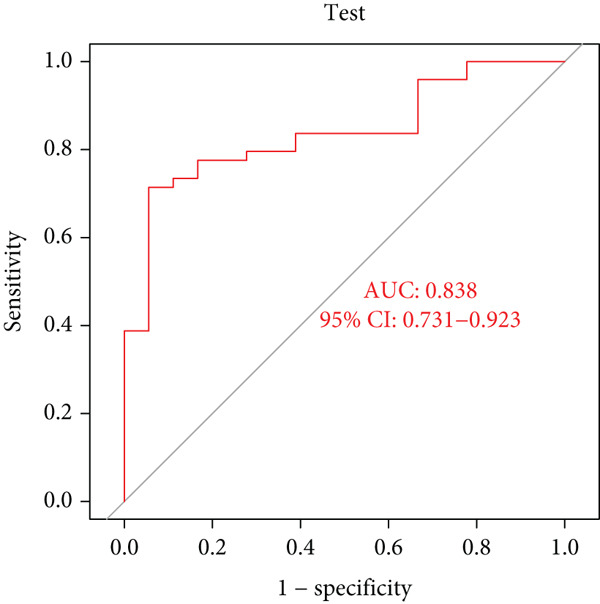
(c)

**Figure:**
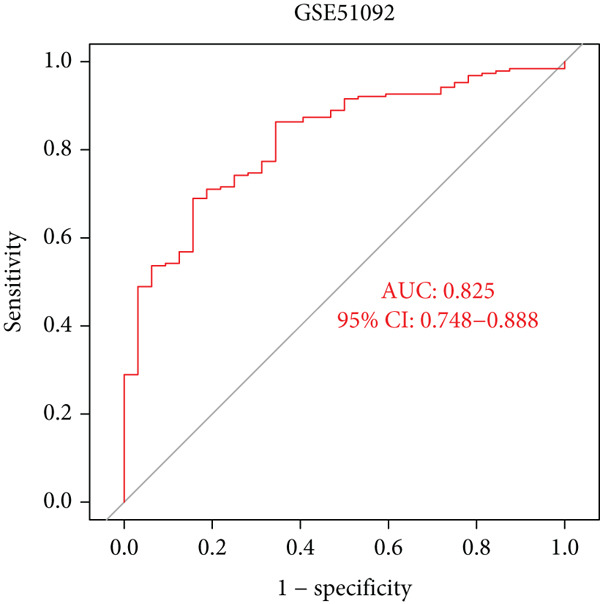
(d)

**Figure:**
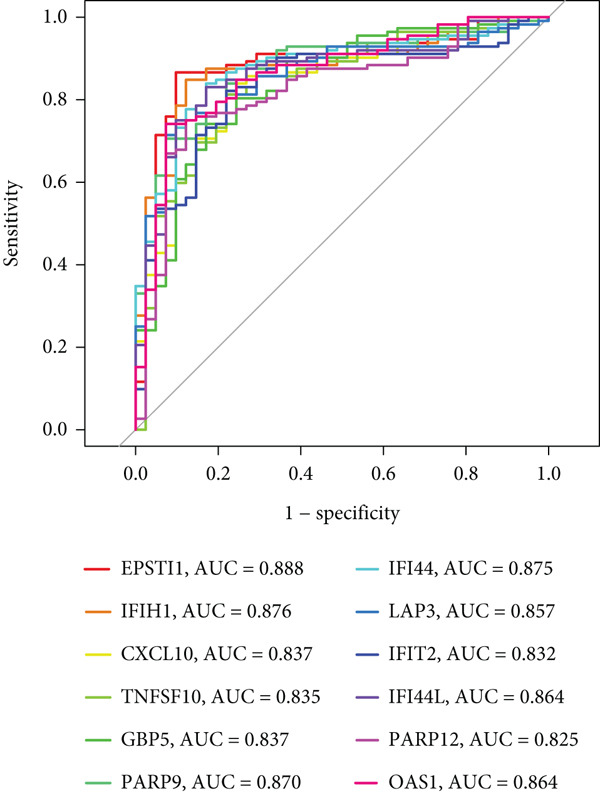
(e)

**Figure:**
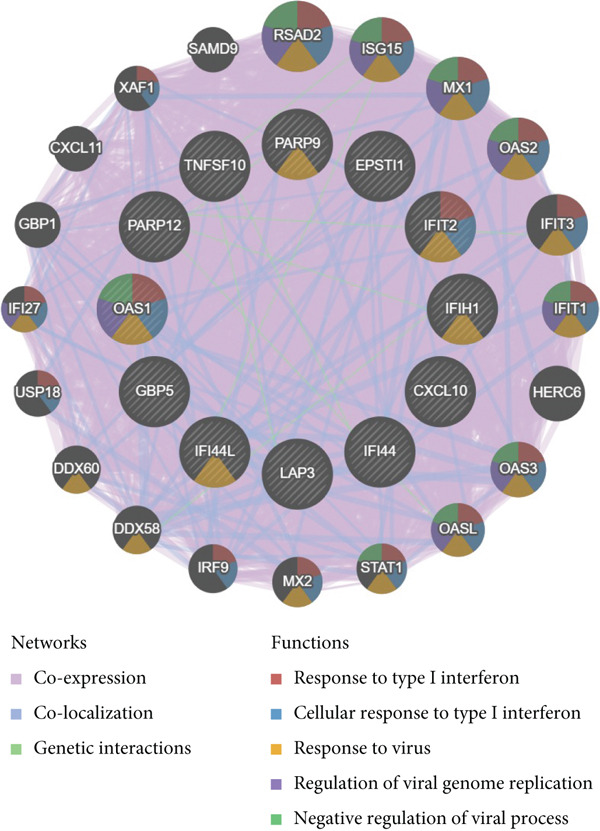
(f)

**Figure:**
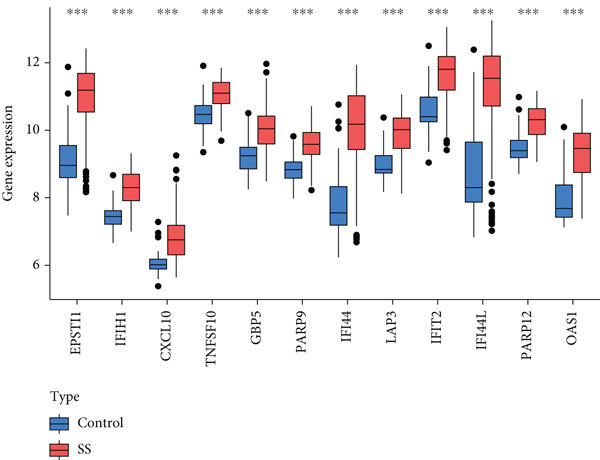
(g)

GeneMANIA network analysis revealed functional interactions among these genes, implicating their roles in Type I interferon response, antiviral immunity, and negative regulation of viral genome replication (Figure 5f), providing mechanistic insights for future exploration.

### 3.6. Correlation Analysis Between Hub Genes and Clinical Indicators

Clinical data including ANA, Ro/SSA, and La/SSB levels were extracted from the GSE84844 dataset. All 12 biomarkers exhibited positive correlations with ANA, Ro/SSA, and La/SSB. Notably, LAP3 showed the strongest association with ANA (*r* = 0.54798, *p* = 0.00172), IFI44L with Ro/SSA (*r* = 0.83312, *p* = 1.11e − 08), and IFI44 with La/SSB (*r* = 0.70709, *p* = 2.59e − 05) (Table 2), reinforcing their clinical relevance in SS diagnosis and disease activity assessment.

**Table 2:** Correlations between key genes and clinical symptoms.

**Gene**	**ANA**		**Ro/SSA**		**La/SSB**	
	**Correlation** **R**	**p** **value**	**Correlation** **R**	**p** **value**	**Correlation** **R**	**p** **value**
CXCL10	0.52049	0.00319	0.66280	6.58e − 05	0.46043	0.01368
EPSTI1	0.51688	0.00345	0.82230	2.49e − 08	0.52765	0.00391
GBP5	0.51012	0.00398	0.62058	0.00025	0.54165	0.00291
IFI44	0.47295	0.00831	0.82878	1.55e − 08	0.57470	0.00138
IFI44L	0.49948	0.00495	0.83312	1.11e − 08	0.62968	0.00033
IFIH1	0.48421	0.00670	0.77338	5.42e − 07	0.49432	0.00750
IFIT2	0.41089	0.02410	0.72783	5.17e − 06	0.57368	0.00141
LAP3	0.54798	0.00172	0.81180	5.20e − 08	0.45091	0.01603
OAS1	0.43968	0.01506	0.72968	4.76e − 06	0.47954	0.00982
PARP12	0.44190	0.01449	0.76855	7.05e − 07	0.58094	0.00119
PARP9	0.37696	0.04003	0.72758	5.22e − 06	0.54081	0.00296
TNFSF10	0.50291	0.00462	0.73065	4.55e − 06	0.70709	2.59e − 05

### 3.7. Immune Cell Infiltration Analysis

CIBERSORT‐based deconvolution of SS peripheral blood transcriptomes revealed significant immune microenvironment remodeling. SS patients exhibited elevated proportions of naive B cells, gamma delta T cells, monocytes, M2 macrophages, and activated dendritic cells, while decreased proportions of memory B cells, CD8+ T cells, regulatory T cells (Tregs), resting NK cells, and M0 macrophages were observed (Wilcoxon rank‐sum test, FDR < 0.05) (Figure 6a). Heat map analysis demonstrated intricate intercellular interactions: naive B cells strongly inversely correlated with memory B cells; gamma delta T cells and Tregs showed negative associations. Monocytes and neutrophils inversely correlated with CD8+ T cells, while gamma delta T cells positively correlated with activated dendritic cells. Resting CD4+ memory T cells exhibited positive associations with monocytes and eosinophils (Figure 6b).

Figure 6Analysis of immune infiltration. (a) Infiltration of different immune cells between Group SS patients and normal controls. The red boxplot corresponds to Group SS patients, and the blue boxplot corresponds to normal controls. (b) Correlation heat map of 22 types of immune cells. Red represents a positive correlation, and blue represents a negative correlation; the darker the color, the stronger the correlation. (c) Associations of SS‐related hub genes with immune cell infiltration.

**Figure:**
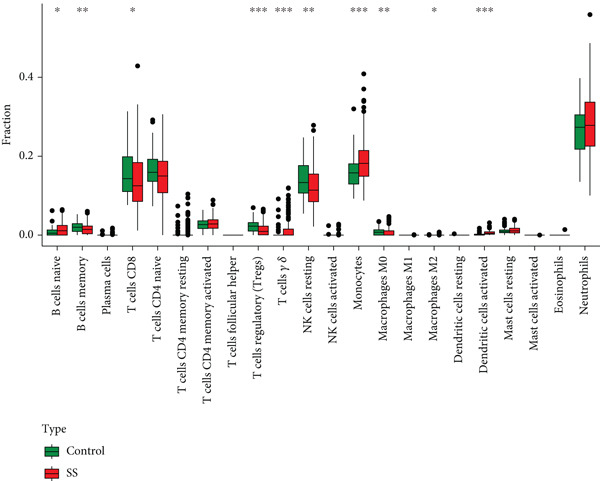
(a)

**Figure:**
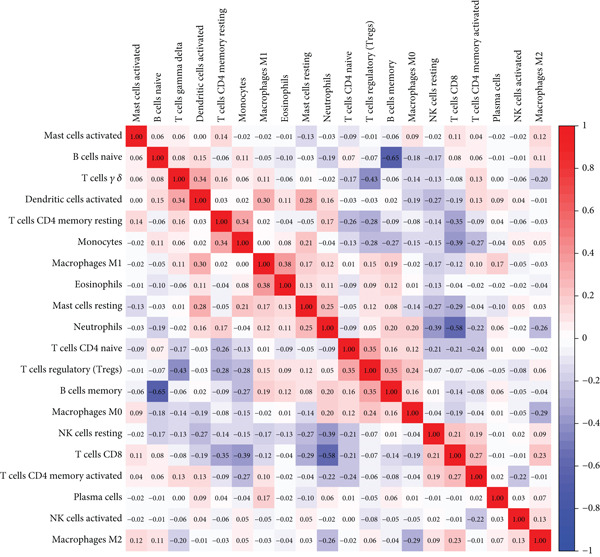
(b)

**Figure:**
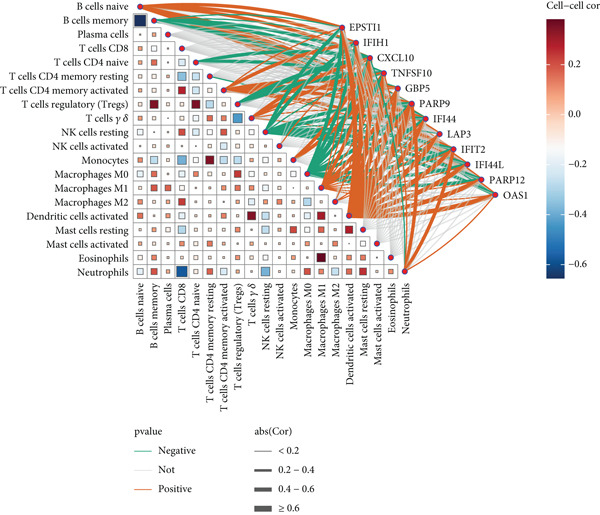
(c)

Notably, the 12 diagnostic biomarkers displayed significant coregulatory relationships with immune subsets. CXCL10 showed the strongest inverse correlation with memory B cells and positive correlation with naive B cells. EPSTI1 correlated most strongly with activated dendritic cells, while CXCL10 inversely associated with M0 macrophages and positively with M1 macrophages. IFIT2 inversely correlated with resting NK cells, and TNFSF10 exhibited negative associations with Tregs (Figure 6c, Supporting Information 2: Table S2), underscoring their roles in immune dysregulation during SS progression.

### 3.8. Single‐Cell Transcriptomic Profiling of PBMCs in SS

To dissect the single‐cell expression landscape of the 12 core genes, we reanalyzed the public dataset GSE157278 (PBMCs from five pSS patients and five healthy controls). After stringent quality control, log‐normalization and Harmony batch correction, 26 transcriptionally distinct clusters were obtained and assigned to eight canonical immune lineages with SingleR (Figure 7a,b). No lineage showed a significant between‐group difference in cellular abundance after multiple‐testing correction (Wilcoxon test, adjusted *p* > 0.05; Figure 7e). Next, we calculated the mean expression of the 12 core genes within each immune subtype. Monocytes and dendritic cells (DCs) displayed the broadest and strongest transcriptional responses, whereas CD4^+^ T and NK cells exhibited a more restricted pattern; all genes remained consistently low in CD8^+^ T cells (Figure 7c). Differential expression between pSS and HC was evaluated with Wilcoxon rank‐sum tests (*F* *D* *R* < 0.05; Figure 7d). The core signature was significantly upregulated in monocytes, DCs, B cells and CD4^+^ T cells. TNFSF10, OAS1 and PARP9 were induced in all seven lineages examined, while GBP5, EPSTI1 and LAP3 were elevated in the majority of them. Conversely, IFIH1 was selectively down‐regulated in NK and T cells, underscoring a lineage‐specific modulation of interferon‐mediated signaling in pSS.

Figure 7Single‐cell transcriptomic profiling of PBMCs in primary Sjögren’s syndrome (pSS). (a) UMAP plot showing the clustering of cells into 26 distinct clusters based on single‐cell RNA sequencing analysis. Each cluster is color‐coded and labeled accordingly. (b) UMAP plot showing the PBMC cell types annotated by SingleR. Colors denote distinct immune cell subsets, including CD4^+^ T cells, T cells, NK cells, CD8^+^T cells, monocytes, B cells, dendritic cells (DCs), and hematopoietic stem cells (HSC). (c) Bubble plot visualizing the average expression (color scale) and percent expressed (dot size) of the 12 diagnostic hub genes across eight major immune cell types. (d) Differential expression heat map of the 12 core genes between pSS and HC groups. Red indicates upregulation in pSS, blue indicates downregulation;  ^∗^ *p* < 0.05,  ^∗∗^ *p* < 0.01, and  ^∗∗∗^ *p* < 0.001 (Wilcoxon test, BH correction). (e) Comparison of immune cell proportions between the pSS and HC groups (Wilcoxon test with BH correction).

**Figure:**
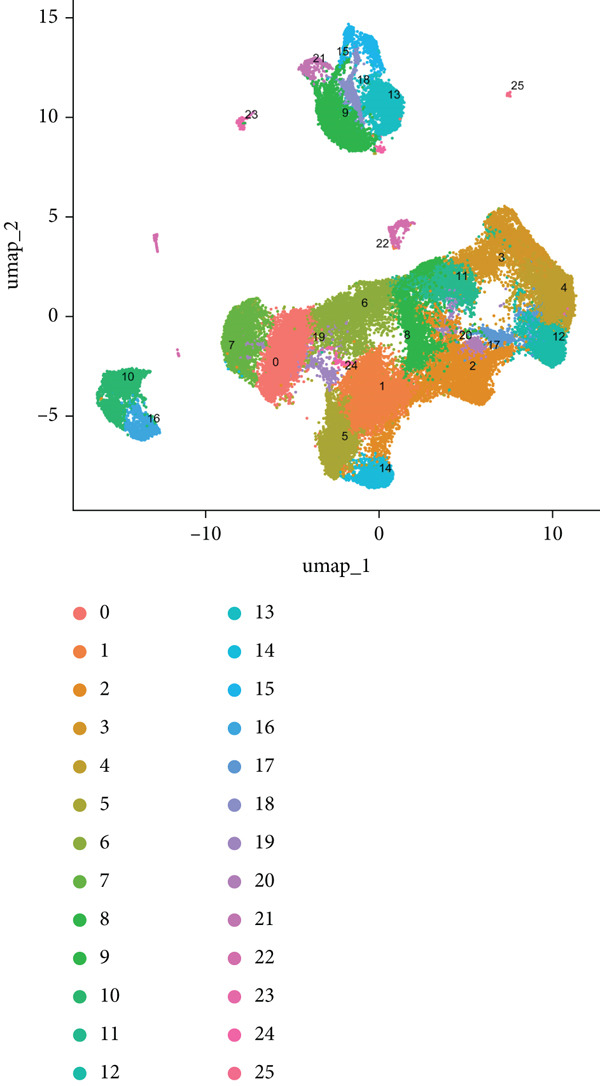
(a)

**Figure:**
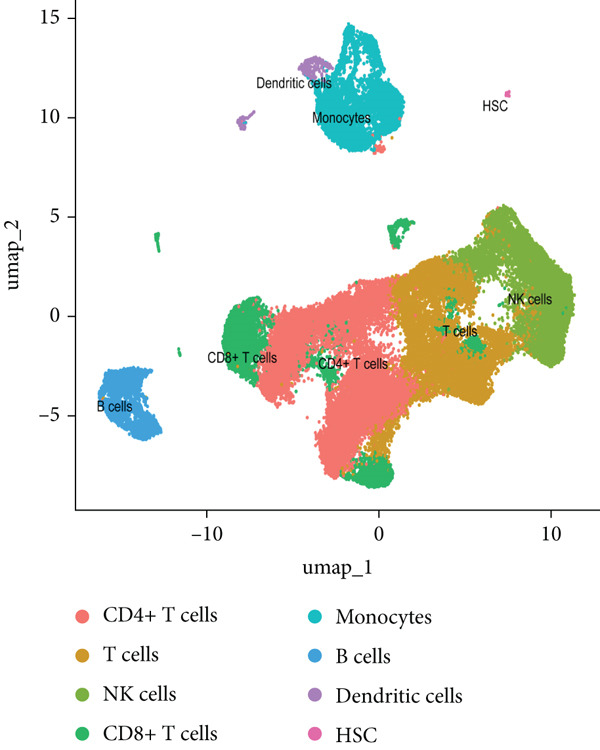
(b)

**Figure:**
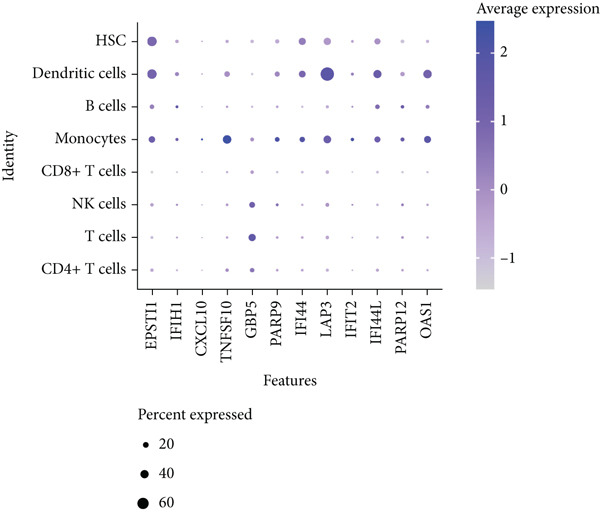
(c)

**Figure:**
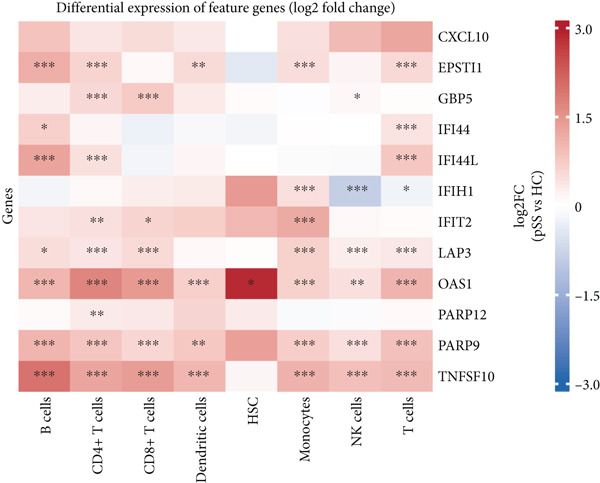
(d)

**Figure:**
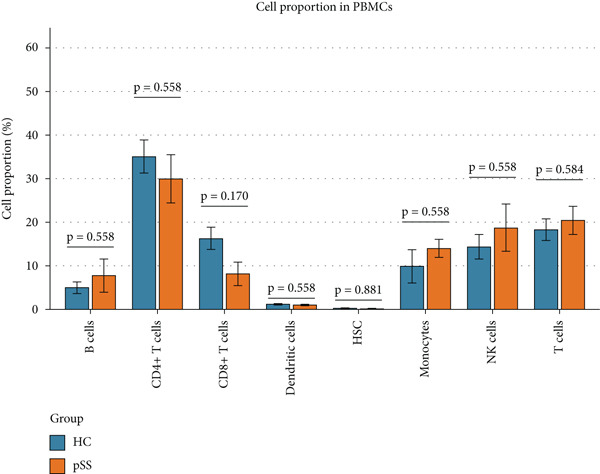
(e)

### 3.9. Virtual Knockdown Analysis Reveals Hub Gene Involvement in Immune Regulatory Networks

**Figure 8:**
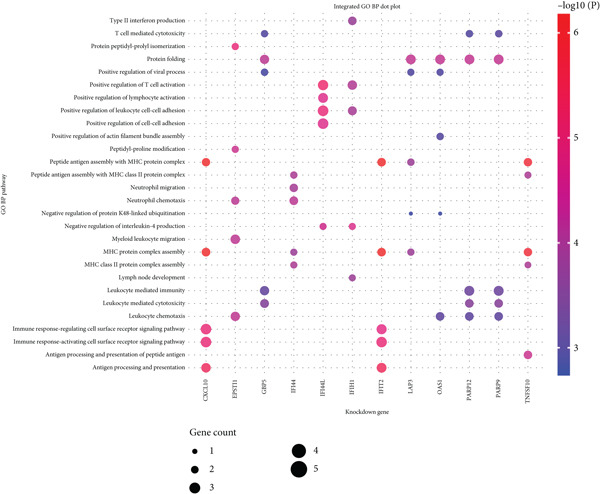
Virtual knockdown analysis reveals the role of core genes in immune regulatory networks. The Top 5 most significant pathways enriched from the Top 20 most perturbed genes following the knockdown of 12 key genes. The *x*‐axis represents the knocked‐down genes, and the *y*‐axis lists the significantly enriched GO BP terms. Dot size indicates the number of genes involved in each BP, and color intensity represents the level of significance.

Virtual knockdown of the 12 hub genes with scTenifoldKnk revealed their differential control of immune‐regulatory circuits in pSS. After each gene was sequentially deleted, edge weights of the residual network were recalculated and perturbed downstream genes were ranked by |*Z* score| × network distance; the Top 20 genes were retained for downstream analysis (Supporting Information 3: Table S3). GO biological‐process enrichment showed that knock‐down of CXCL10, IFIT2, or TNFSF10 selectively impaired antigen processing and presentation; IFIH1 deletion specifically disrupted type‐II interferon production; IFI44L loss altered T‐cell activation and lymphocyte regulation; LAP3, OAS1, PARP9, or GBP5 silencing disturbed protein folding and modification; whereas EPSTI1 or IFI44 knock‐down markedly dysregulated leukocyte migration, including neutrophil chemotaxis. These data indicate that, although functionally interconnected, individual pSS hub genes govern distinct immune modules—antigen presentation, interferon signaling, proteostasis and leukocyte trafficking—that collectively maintain immune network homeostasis (Figure 8).

### 3.10. Screening of Potential Therapeutic Agents

The L1000 FWD platform was employed to identify candidate drugs targeting the 12 hub genes. BRD‐K66037923 and GW‐405833 demonstrated the most prominent gene expression reversal effects. Notably, FDA‐approved drugs Nisoldipine and Exemestane (similarity score = −0.7778) were identified as potential repurposing candidates (Table 3), offering novel therapeutic avenues for SS management.

**Table 3:** Small molecule drugs identified via L1000 FWD using 12 key genes.

**Sig_id**	**Drug**	**Similarity score**	**p** **value**	**q** **value**	**Z** **score**	**Combined score**
CPC008_PC3_24H:BRD‐K66037923‐001‐04‐4:10	BRD‐K66037923	−0.8889	1.19e − 13	1.06e − 10	1.75	−22.57
CPC006_HT29_24H:BRD‐K10705233‐003‐02‐8:40	GW‐405833	−0.8889	1.13e − 13	1.06e − 10	1.79	−23.23
CPC008_HCC515_6H:BRD‐K75999307‐001‐05‐7:10	BRD‐K75999307	−0.7778	1.52e − 11	9.03e − 09	1.77	−19.16
CPC006_NCIH508_6H:BRD‐K33583600‐001‐09‐6:10	Isoliquiritigenin	−0.7778	1.19e − 11	7.71e − 09	1.82	−19.9
CPC011_PC3_24H:BRD‐A96107863‐001‐10‐0:10	Nisoldipine	−0.7778	3.05e − 11	1.23e − 08	1.71	−17.97
CPC008_PC3_6H:BRD‐K66037923‐001‐04‐4:10	BRD‐K66037923	−0.7778	2.34e − 11	1.08e − 08	1.76	−18.68
CPC013_HEPG2_6H:BRD‐A95820578‐001‐01‐4:10	BRD‐A95820578	−0.7778	2.76e − 11	1.17e − 08	1.69	−17.89
CPC005_VCAP_24H:BRD‐A82238138‐001‐01‐7:10	Budesonide	−0.7778	2.27e − 11	1.06e − 08	1.81	−19.23
CPC001_VCAP_6H:BRD‐A10355991‐003‐01‐8:10	Norketamine	−0.6667	1.81e − 09	4.97e − 07	1.84	−16.05
CPC004_HCC515_24H:BRD‐A73741725‐001‐01‐0:10	Exemestane	−0.6667	1.32e − 09	4.92e − 07	1.86	−16.48

## 4. Discussion

SS is a chronic autoimmune disease whose diagnosis has long relied on serological antibodies such as anti‐Ro/SSA and anti‐La/SSB. In the 2016 classification criteria for pSS, anti‐SSA antibodies were designated as the sole serological biomarker with definitive diagnostic value [30]. However, approximately one‐third of SS patients are seronegative, and the presence of anti‐SSA antibodies often indicates systemic immune dysregulation and a relatively advanced disease stage. Consequently, reliance on conventional antibody testing alone can easily lead to underdiagnosis in patients with early‐stage disease or those who are seronegative [31, 32]. To address this critical limitation, we integrated multicenter peripheral blood transcriptomic datasets and systematically screened and validated candidates within a ML framework encompassing 113 algorithm combinations. We ultimately established a diagnostic model based on 12 key genes (EPSTI1, IFIH1, CXCL10, TNFSF10, GBP5, PARP9, IFI44, LAP3, IFIT2, IFI44L, PARP12, and OAS1). This model demonstrated excellent diagnostic performance: it achieved an AUC of 0.994 (mean AUC 0.885) in the training set, and an overall sensitivity of 70.7% and specificity of 84.0% in the combined validation sets. Its efficacy surpasses that of other recently reported blood transcriptome‐based diagnostic models (AUC 0.822–0.903) [33–36]. Notably, it can provide molecular‐level objective evidence for patients who are seronegative but exhibit highly suggestive clinical manifestations of SS, thereby serving as a crucial complement to existing serological tests.

Within the current diagnostic pathway for SS, labial gland biopsy, while capable of providing key pathological evidence, is limited in widespread screening applications due to its invasiveness, potential for sampling error, and low patient acceptance. In recent years, saliva has emerged as an ideal medium for investigating local pathological changes in SS owing to its non‐invasive collection and operational simplicity. Numerous proteomic studies have identified aberrant protein expression in the saliva of pSS patients, such as *β*2‐microglobulin, carbonic anhydrase VI, neutrophil gelatinase‐associated lipocalin, and calprotectin. These alterations are closely associated with local chronic inflammation, abnormal immunoglobulin secretion, and glandular hypofunction [37]. However, salivary protein biomarkers overall possess limited diagnostic specificity, are susceptible to influences from salivary flow rate, collection methods, and local oral conditions, and their standardization processes remain incomplete, hindering multicenter application. In contrast, the peripheral blood RNA testing workflow employed in our study is more readily standardized across centers and subjected to systematic quality control, offering good reproducibility and clinical applicability. From a pathophysiological perspective, blood transcriptomic markers primarily reflect systemic immune dysregulation, whereas salivary proteins more directly indicate local glandular inflammation and tissue damage, thus providing complementary information from “systemic” and “local” vantage points, respectively. Future integration of these two marker types holds promise for constructing a dual‐track diagnostic strategy to more comprehensively capture disease features.

Based on its performance characteristics and technical advantages, this 12‐gene model demonstrates potential for various clinical applications. For disease diagnosis, it can aid in identifying seronegative SS and facilitating early diagnosis. Regarding disease monitoring, the expression of key genes such as IFI44L and LAP3 significantly correlates with clinical indicators of disease activity. Coupled with standardized detection platforms like qPCR for rapid quantitative analysis, it can provide molecular evidence for dynamic disease changes. In terms of treatment stratification, this gene set serves as a core surrogate indicator for type I interferon pathway activation. Following validation in prospective clinical studies, it could potentially help identify suitable candidates for targeted therapies like JAK inhibitors, thereby advancing SS treatment from empirical to precision medicine.

Concurrently, the biological functions of these diagnostic markers provide strong support for the “viral mimicry” hypothesis in SS, which posits that endogenous nucleic acids can trigger innate immune pathways typically reserved for viral pathogens. WGCNA and functional enrichment analyses revealed that the hub genes are significantly enriched in antiviral immune‐related pathways, including influenza A, hepatitis C, COVID‐19, and RIG‐I‐like receptor signaling pathways. The upregulation of IFIH1 (MDA5), a key member of the RIG‐I‐like receptor family, further suggests an important role for innate immune responses triggered by endogenous aberrant nucleic acids in SS pathogenesis [38, 39]. Furthermore, as interferon‐stimulated genes (ISGs), IFI44 and IFI44L are not only closely associated with the sustained activation of the Type I interferon signaling pathway but their expression levels also show significant positive correlations with anti‐Ro/SSA and anti‐La/SSB antibody titers, highlighting their pivotal bridging role in connecting innate immune abnormalities to autoantibody production [40].

To investigate the impact of these genes on the immune microenvironment, we observed significant immune remodeling in the peripheral blood of SS patients, characterized by increased proportions of naïve B cells, gamma delta T (*γδ* T) cells, monocytes, M2 macrophages, and activated dendritic cells, alongside decreased proportions of memory B cells, CD8^+^ T cells, Tregs, and resting NK cells. The 12 core genes exhibit extensive co‐regulatory relationships with these immune cell subset alterations: for instance, CXCL10 showed the strongest negative correlation with memory B cells, a positive correlation with naïve B cells, and actively promoted monocyte infiltration, potentially playing a central role in pro‐inflammatory microenvironment formation and glandular damage [41–43]; TNFSF10 (TRAIL) correlated positively with *γδ* T cells and negatively with Tregs, suggesting its involvement in tissue damage possibly through modulating immune cell balance [44, 45]; EPSTI1 correlated positively with naïve B cells, potentially promoting autoantibody production via pathways like STAT1 [46–48]; PARP9 correlated negatively with memory B cells, suggesting it might interfere with normal B cell differentiation trajectories, thereby disrupting immune tolerance [49, 50]. Additionally, the positive correlation between CXCL10 and M2 macrophages suggests its potential involvement in mediating glandular fibrosis progression via the TGF‐*β* signaling pathway [51, 52].

To validate the expression of these hub genes at single‐cell resolution and pinpoint their cellular localization, we analyzed single‐cell transcriptomic data from PBMCs. Single‐cell analysis revealed their most prominent expression in monocytes and dendritic cells, which exhibited the broadest and strongest transcriptional responses, with significant upregulation also observed in B cells and CD4^+^ T cells in pSS patients, although CD4^+^ T cells showed a more restricted expression pattern. To further explore the potential functional roles of these hub genes, we performed in silico knockdown analyses. The results indicated that they play distinct and specialized roles within the immune regulatory network: for example, knockdown of CXCL10, IFIT2, or TNFSF10 primarily affected antigen processing and presentation; IFIH1 knockdown specifically perturbed type II interferon production; whereas knockdown of EPSTI1 or IFI44 significantly disrupted leukocyte migration and neutrophil chemotaxis. These findings suggest that the 12 hub genes collectively regulate key immune modules, including antigen presentation, interferon signaling, and leukocyte trafficking, and their synergistic dysregulation collaboratively promotes the immunopathological process of SS.

Building upon these mechanistic insights, we further utilized the L1000FWD platform for drug repositioning screening, identifying several compounds with the potential to reverse the hub gene expression signature. Among them, the FDA‐approved drugs Nisoldipine and Exemestane are particularly noteworthy. The identification of Exemestane, an aromatase inhibitor, aligns with the marked female predominance of SS [5, 53], providing new clues for exploring the role of sex hormone metabolism in SS autoimmune dysregulation.

Despite identifying a promising diagnostic model and therapeutic candidates, the limitations of our study warrant careful consideration. First, all analyses are based on transcriptomic data from public databases; the protein‐level expression and clinical applicability of these biomarkers require further confirmation. Although cross‐cohort validation was performed, the cohort populations were limited to specific geographical regions, restricting generalizability to other ethnic groups. Furthermore, while the specific molecular mechanisms of the hub genes were predicted via in silico knockdown, they ultimately require validation through in vitro and in vivo experiments. Finally, the therapeutic potential of the computationally identified candidate drugs must be substantiated by subsequent empirical studies. Corresponding future research will focus on: validating this diagnostic model in prospective multicenter trials; gaining deeper insights into pathogenesis through multi‐omics integration; promoting the development of clinically applicable detection technologies; and elucidating the biological roles of key genes through functional experiments.

In summary, by integrating multi‐omics data and large‐scale ML analysis, we have developed and validated a high‐performance 12‐gene diagnostic model. This model not only demonstrates significant potential for clinical translation but also deepens the understanding of SS immunopathological mechanisms, particularly the link between the “viral mimicry” hypothesis and the dysregulation of specific immune modules. Future research should focus on validating this model in prospective cohorts, developing clinically suitable detection assays, and further exploring the biological functions of the hub genes and the therapeutic potential of the candidate drugs, thereby advancing SS diagnosis and treatment towards precision and personalized medicine.

## Ethics Statement

The authors have nothing to report.

## Consent

The authors have nothing to report.

## Conflicts of Interest

The authors declare no conflicts of interest.

## Author Contributions

H.X. and J.C. designed the study. J.C. performed the data collection. H.X., Y.S., and Y.Z. analyzed and interpreted the data. Y.L., Y.G., and H.J. contributed to the single‐cell data analysis and computational virtual knockdown analysis. D.Z. and X.C. participated in the manuscript proofreading. H.X. drafted the manuscript, and J.C. revised the manuscript.

## Funding

No funding was received for this manuscript.

## Supporting Information

Additional supporting information can be found online in the Supporting Information section.

## Supporting information


**Supporting Information 1** Table S1: Classification performance under the unified threshold.

**Supporting Information 2** Table S2: Correlation analysis between 12 diagnostic biomarkers and immune subsets in Sjögren’s syndrome (*p*.val < 0.05).

**Supporting Information 3** Table S3: Significant genes Top 20.

**Supporting Information 4** Figure S1: Quality‐control violin plots of single‐cell metrics, showing nFeature_RNA, nCount_RNA, percent.mt, percent.hb, and percent.platelet. Figure S2: UMAP visualization of cell types stratified by group (HC vs pSS). Colors denote immune‐cell identities; HC cohort is displayed on the left and pSS cohort on the right.

## Data Availability

The publicly available datasets analyzed in this study (GSE51092, GSE66795, GSE84844, and GSE157278) can be found in the GEO database.
