# Supplementary material for: Integrative Bioinformatics and Machine Learning Identify Novel Diagnostic Biomarkers and Molecular Mechanisms in Sjögren’s Syndrome
Source: Int J Genomics. 2026 Jan 16;2026:5044551. doi: 10.1155/ijog/5044551 (PMC12811409; doi:10.1155/ijog/5044551)
Supplement: Supplementary file 4 — Supporting Information 4 Figure S1: Quality‐control violin plots of single‐cell metrics, showing nFeature_RNA, nCount_RNA, percent.mt, percent.hb, and percent.platelet. Figure S2: UMAP visualization of cell types stratified by group (HC vs pSS). Colors denote immune‐cell identities; HC cohort is displayed on the left and pSS cohort on the right. [file IJOG-2026-5044551-s004.pdf]

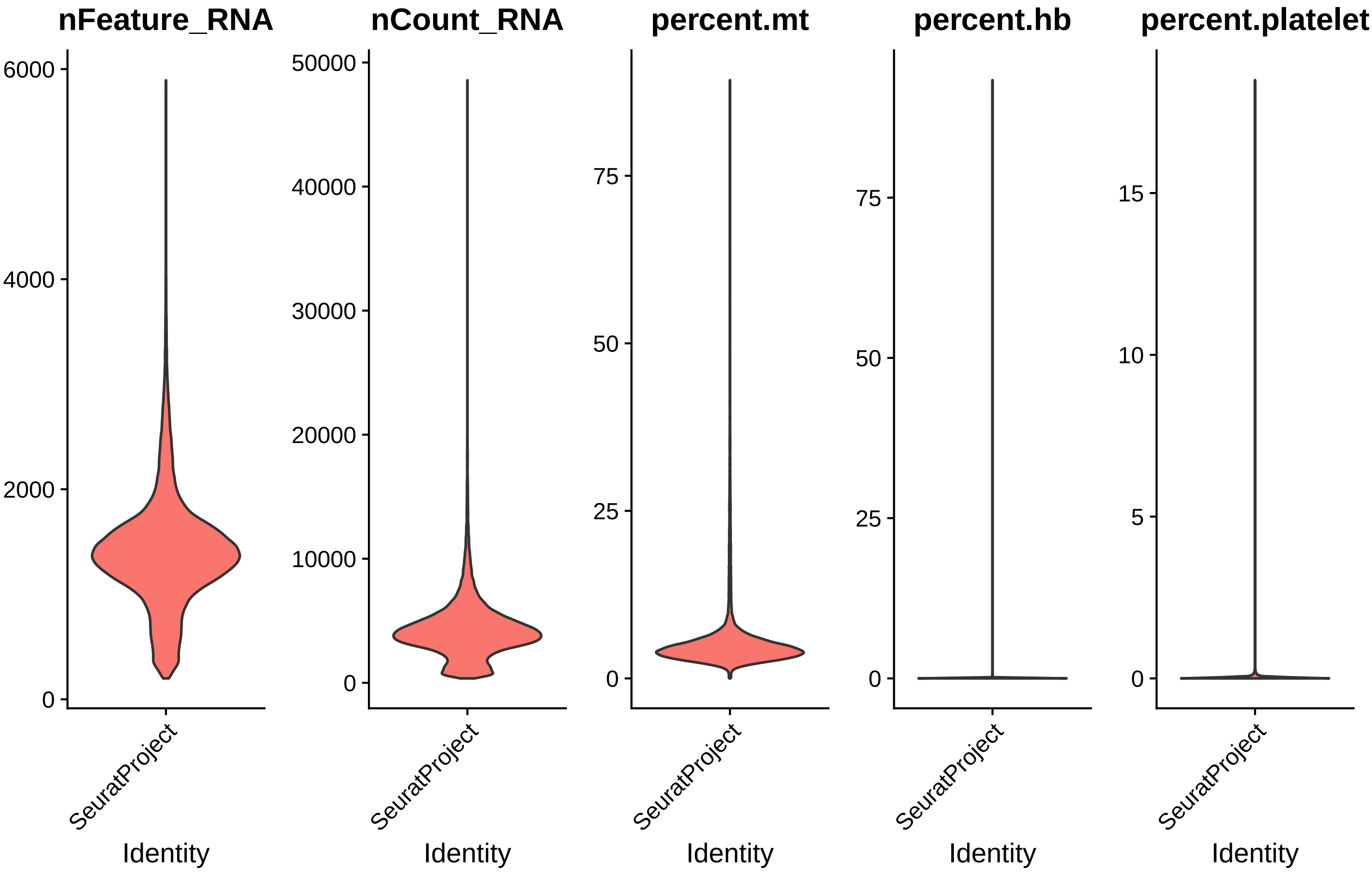


**Fig. S1** Quality-control violin plots of single-cell metrics, showing nFeature_RNA, nCount_RNA, percent.mt, percent.hb and percent.platelet.

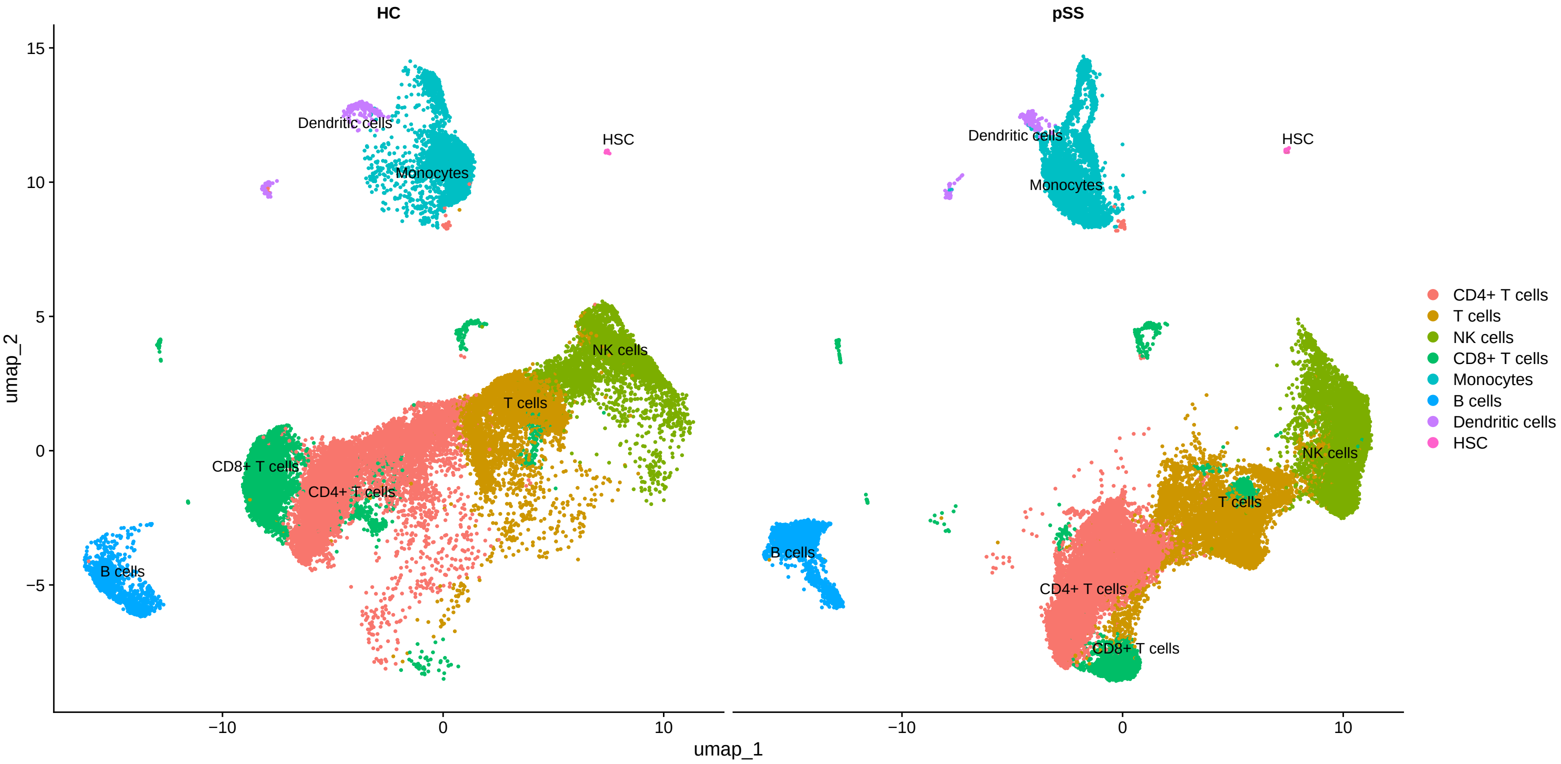


**Fig. S2** UMAP visualization of cell types stratified by group (HC vs pSS). Colors denote immune-cell identities; HC cohort is displayed on the left and pSS cohort on the right.
